# Di-*n*-but­yl{4-hydr­oxy-*N*′-[(2-oxido-1-naphthyl-κ*O*)methyl­ene]benzo­hydrazidato-κ^2^ *O*,*N*′}tin(IV)

**DOI:** 10.1107/S1600536810014662

**Published:** 2010-04-24

**Authors:** Md. Abu Affan, Norrihan Sam, Fasihuddin Ahmad, Seik Weng Ng

**Affiliations:** a Faculty of Resource Science and Technology, Universiti Malaysia Sarawak, 94300 Kota Samarahan, Sarawak, Malaysia; b Department of Chemistry, University of Malaya, 50603 Kuala Lumpur, Malaysia

## Abstract

The deprotonated Schiff base ligand in the title compound, [Sn(C_4_H_9_)_2_(C_18_H_12_N_2_O_3_)], *O*,*N*,*O*′-chelates to the Sn atom, which is five-coordinated in a *cis*-C_2_NO_2_Sn trigonal-bipyramidal environment. The apical sites are occupied by the O atoms [O—Sn—O = 155.2 (2)°]. The hydr­oxy group is a hydrogen-bond donor to the two-coordinate N atom of an adjacent mol­ecule, the hydrogen-bonding inter­action giving rise to a helical chain running along the *c* axis. The carbon atoms of the butyl chains are equally disordered over two positions.

## Related literature

For the synthesis of the Schiff base, see: Cui *et al.* (2007 ▶).
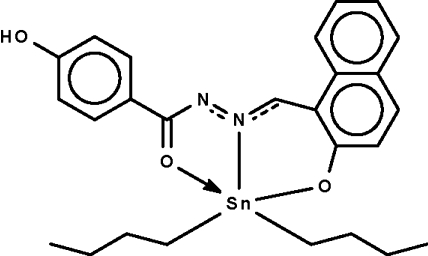


## Experimental

### 

#### Crystal data

[Sn(C_4_H_9_)_2_(C_18_H_12_N_2_O_3_)]
*M* *_r_* = 537.21
Monoclinic, 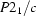

*a* = 11.6644 (9) Å
*b* = 17.2500 (14) Å
*c* = 12.9296 (11) Å
β = 106.793 (1)°
*V* = 2490.6 (4) Å^3^
*Z* = 4
Mo *K*α radiation
μ = 1.05 mm^−1^
*T* = 293 K
0.40 × 0.40 × 0.10 mm

#### Data collection

Bruker SMART APEX diffractometer
Absorption correction: multi-scan (*SADABS*; Sheldrick, 1996 ▶) *T*_min_ = 0.678, *T*_max_ = 0.902
19289 measured reflections
4391 independent reflections
2953 reflections with *I* > 2σ(*I*)
*R*_int_ = 0.038

#### Refinement

*R*[*F* ^2^ > 2σ(*F* ^2^)] = 0.057
*wR*(*F* ^2^) = 0.189
*S* = 1.03
4391 reflections
314 parameters
78 restraints
H-atom parameters constrained
Δρ_max_ = 1.19 e Å^−3^
Δρ_min_ = −1.14 e Å^−3^

### 

Data collection: *APEX2* (Bruker, 2009 ▶); cell refinement: *SAINT* (Bruker, 2009 ▶); data reduction: *SAINT*; program(s) used to solve structure: *SHELXS97* (Sheldrick, 2008 ▶); program(s) used to refine structure: *SHELXL97* (Sheldrick, 2008 ▶); molecular graphics: *X-SEED* (Barbour, 2001 ▶); software used to prepare material for publication: *publCIF* (Westrip, 2010 ▶).

## Supplementary Material

Crystal structure: contains datablocks global, I. DOI: 10.1107/S1600536810014662/bt5252sup1.cif

Structure factors: contains datablocks I. DOI: 10.1107/S1600536810014662/bt5252Isup2.hkl

Additional supplementary materials:  crystallographic information; 3D view; checkCIF report

## Figures and Tables

**Table 1:** Hydrogen-bond geometry (Å, °)

*D*—H⋯*A*	*D*—H	H⋯*A*	*D*⋯*A*	*D*—H⋯*A*
O3—H3⋯N2^i^	0.84	1.99	2.824 (6)	177

Symmetry code: (i) 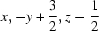.

**Fig. 1.**
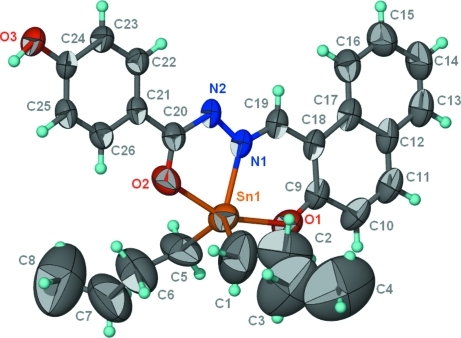
Anisotropic displacement ellipsoid plot (Barbour, 2001) of Sn(C4H9)2(C18H12N2O3) at the 50% probability level; hydrogen atoms are drawn as spheres of arbitrary radius. The disorder in the butyl chains is not shown.

## supplementary crystallographic information

### Comment

There are numerous reports of metal complexes of the Schiff bases derived by condensing salicylaldehyde (and related compounds) with benzohydrazide (and related compounds). *E*)-4-Hydroxy-*N*'-[(2-hydroxynaphthalen-1-yl)methylene]benzohydrazide is a less studied ligand. The deprotonated Schiff-base ligand in the title compound *O*,*N*,*O*'-chelates to the tin atom, which is five coordinate in a *cis*-C_2_NO_2_Sn trigonal bipyramidal environment (Scheme I, Fig. 1). The apical sites are occupied by the oxgen atoms [O–Sn–O 155.2 (2) °]. Both butyl chains are disordered over two positions in a 1:1 ratio.

### Experimental

(*E*)-4-Hydroxy-*N*'-[(2-hydroxynaphthalen-1-yl)methylene]benzohydrazide was synthesized according to a literature method (Cui *et al.*, 2007). The compound (0.61 g, 2 mmole) was dissolved in methanol (20 ml). Potassium hydroxide (0.23 g, 4 mmol) dissolved in methanol (5 ml) was added. The orange solution was then treated with di-*n*-butyltin dichloride (0.61 g, 2 mmol) in methanol (10 ml). The mixture was heated for an hour. The solution was filtered. The evaporation of the solvent gave a product that was recrystallized from ether in 70% yield; m.p. 455-457 K.

### Refinement

Hydrogen atoms were placed in calculated positions (C—H 0.93 to 0.97, O–H 0.84 Å) and were included in the refinement in the riding model approximation, with *U*(H) set to 1.2 to 1.5*U*_eq_(C,*O*).

The carbon atoms of the butyl chains are disordered over two positions; the occupancy could not be refined, and was assumed to be 50:50. The 1,2-related carbon-carbon distances were restrained to 1.54±0.01Å and the 1,3-related ones to 2.51±0.01 Å. The displacement ellipsoids of the primed atoms were restrained to be similar  to those of the unprimed ones; furthermore, the ADP's were restrained to be nearly isotropic.

The final difference Fourier map had a peak/hole in the vicinity of Sn1.

### Crystal data

**Table:**

[Sn(C_4_H_9_)_2_(C_18_H_12_N_2_O_3_)]	*F*(000) = 1096
*M**_r_* = 537.21	*D*_x_ = 1.433 Mg m^−^^3^
Monoclinic, *P*2_1_/*c*	Mo *K*α radiation, λ = 0.71073 Å
Hall symbol: -P 2ybc	Cell parameters from 4338 reflections
*a* = 11.6644 (9) Å	θ = 2.2–21.2°
*b* = 17.2500 (14) Å	µ = 1.05 mm^−^^1^
*c* = 12.9296 (11) Å	*T* = 293 K
β = 106.793 (1)°	Plate, orange
*V* = 2490.6 (4) Å^3^	0.40 × 0.40 × 0.10 mm
*Z* = 4	

### Data collection

**Table:**

Bruker SMART APEX diffractometer	4391 independent reflections
Radiation source: fine-focus sealed tube	2953 reflections with *I* > 2σ(*I*)
graphite	*R*_int_ = 0.038
ω scans	θ_max_ = 25.0°, θ_min_ = 1.8°
Absorption correction: multi-scan (*SADABS*; Sheldrick, 1996)	*h* = −13→13
*T*_min_ = 0.678, *T*_max_ = 0.902	*k* = −20→20
19289 measured reflections	*l* = −15→15

### Refinement

**Table:**

Refinement on *F*^2^	Primary atom site location: structure-invariant direct methods
Least-squares matrix: full	Secondary atom site location: difference Fourier map
*R*[*F*^2^ > 2σ(*F*^2^)] = 0.057	Hydrogen site location: inferred from neighbouring sites
*wR*(*F*^2^) = 0.189	H-atom parameters constrained
*S* = 1.03	*w* = 1/[σ^2^(*F*_o_^2^) + (0.0911*P*)^2^ + 4.703*P*] where *P* = (*F*_o_^2^ + 2*F*_c_^2^)/3
4391 reflections	(Δ/σ)_max_ = 0.001
314 parameters	Δρ_max_ = 1.19 e Å^−^^3^
78 restraints	Δρ_min_ = −1.14 e Å^−^^3^

### Fractional atomic coordinates and isotropic or equivalent isotropic displacement parameters (Å^2^)

**Table:**

	*x*	*y*	*z*	*U*_iso_*/*U*_eq_	Occ. (<1)
Sn1	0.23178 (5)	0.55623 (3)	0.50133 (4)	0.0893 (3)	
O1	0.2190 (5)	0.4955 (4)	0.6362 (5)	0.1033 (17)	
O2	0.3235 (5)	0.6123 (3)	0.4016 (4)	0.0965 (16)	
O3	0.6606 (4)	0.8107 (3)	0.2051 (3)	0.0807 (13)	
H3	0.6096	0.8344	0.1560	0.121*	
N1	0.4181 (5)	0.5601 (2)	0.5908 (4)	0.0602 (12)	
N2	0.4920 (4)	0.6041 (3)	0.5446 (3)	0.0602 (12)	
C1	0.100 (2)	0.6351 (12)	0.4946 (19)	0.145 (6)	0.50
H1A	0.0795	0.6612	0.4253	0.174*	0.50
H1B	0.0285	0.6080	0.4998	0.174*	0.50
C2	0.1348 (19)	0.6952 (16)	0.584 (2)	0.161 (7)	0.50
H2A	0.2007	0.7262	0.5752	0.193*	0.50
H2B	0.1611	0.6695	0.6536	0.193*	0.50
C3	0.026 (2)	0.7481 (16)	0.5794 (19)	0.188 (7)	0.50
H3A	0.0199	0.7890	0.5266	0.226*	0.50
H3B	−0.0477	0.7180	0.5585	0.226*	0.50
C4	0.044 (3)	0.7835 (19)	0.693 (2)	0.212 (9)	0.50
H4A	−0.0208	0.8182	0.6915	0.319*	0.50
H4B	0.1183	0.8114	0.7143	0.319*	0.50
H4C	0.0457	0.7427	0.7441	0.319*	0.50
C5	0.143 (2)	0.4749 (16)	0.3952 (13)	0.108 (5)	0.50
H5A	0.1820	0.4252	0.4169	0.130*	0.50
H5B	0.0625	0.4710	0.4008	0.130*	0.50
C6	0.136 (2)	0.4884 (13)	0.2768 (13)	0.134 (6)	0.50
H6A	0.2165	0.4907	0.2693	0.161*	0.50
H6B	0.0976	0.5378	0.2536	0.161*	0.50
C7	0.066 (3)	0.4238 (13)	0.2044 (14)	0.171 (7)	0.50
H7A	−0.0101	0.4156	0.2194	0.205*	0.50
H7B	0.1109	0.3758	0.2183	0.205*	0.50
C8	0.044 (3)	0.4484 (17)	0.0853 (13)	0.189 (7)	0.50
H8A	0.0044	0.4072	0.0387	0.284*	0.50
H8B	0.1196	0.4592	0.0723	0.284*	0.50
H8C	−0.0049	0.4941	0.0711	0.284*	0.50
C1'	0.157 (2)	0.6678 (14)	0.5198 (18)	0.145 (6)	0.50
H1'A	0.2129	0.7077	0.5135	0.174*	0.50
H1'B	0.0842	0.6750	0.4606	0.174*	0.50
C2'	0.127 (2)	0.6797 (14)	0.6253 (18)	0.161 (7)	0.50
H2'A	0.1957	0.6673	0.6857	0.193*	0.50
H2'B	0.0614	0.6457	0.6282	0.193*	0.50
C3'	0.090 (2)	0.7660 (13)	0.633 (3)	0.188 (7)	0.50
H3'A	0.1350	0.7881	0.7012	0.226*	0.50
H3'B	0.1062	0.7960	0.5751	0.226*	0.50
C4'	−0.044 (2)	0.7688 (18)	0.622 (3)	0.212 (9)	0.50
H4'A	−0.0677	0.8215	0.6277	0.319*	0.50
H4'B	−0.0596	0.7387	0.6792	0.319*	0.50
H4'C	−0.0882	0.7478	0.5537	0.319*	0.50
C5'	0.175 (2)	0.4609 (16)	0.3742 (15)	0.108 (5)	0.50
H5'A	0.2417	0.4426	0.3507	0.130*	0.50
H5'B	0.1387	0.4175	0.4009	0.130*	0.50
C6'	0.082 (2)	0.5037 (12)	0.2830 (16)	0.134 (6)	0.50
H6'A	0.0244	0.5292	0.3122	0.161*	0.50
H6'B	0.1227	0.5433	0.2530	0.161*	0.50
C7'	0.0180 (18)	0.4479 (17)	0.1940 (18)	0.171 (7)	0.50
H7'A	−0.0441	0.4754	0.1404	0.205*	0.50
H7'B	−0.0196	0.4072	0.2244	0.205*	0.50
C8'	0.107 (3)	0.4120 (18)	0.140 (2)	0.189 (7)	0.50
H8'A	0.0653	0.3760	0.0852	0.284*	0.50
H8'B	0.1687	0.3852	0.1932	0.284*	0.50
H8'C	0.1418	0.4522	0.1077	0.284*	0.50
C9	0.2968 (7)	0.4704 (4)	0.7234 (6)	0.0758 (18)	
C10	0.2491 (8)	0.4253 (5)	0.7944 (7)	0.095 (2)	
H10	0.1672	0.4159	0.7764	0.114*	
C11	0.3217 (8)	0.3965 (4)	0.8869 (7)	0.095 (2)	
H11	0.2880	0.3687	0.9324	0.113*	
C12	0.4480 (7)	0.4071 (4)	0.9177 (5)	0.0766 (19)	
C13	0.5209 (9)	0.3732 (4)	1.0119 (6)	0.093 (2)	
H13	0.4861	0.3451	1.0564	0.111*	
C14	0.6389 (10)	0.3806 (5)	1.0389 (6)	0.104 (3)	
H14	0.6868	0.3583	1.1022	0.124*	
C15	0.6900 (9)	0.4220 (6)	0.9718 (7)	0.108 (3)	
H15	0.7728	0.4269	0.9906	0.130*	
C16	0.6221 (8)	0.4556 (4)	0.8790 (6)	0.086 (2)	
H16	0.6591	0.4829	0.8355	0.104*	
C17	0.4985 (7)	0.4495 (3)	0.8489 (5)	0.0700 (17)	
C18	0.4202 (6)	0.4832 (3)	0.7504 (5)	0.0631 (15)	
C19	0.4717 (6)	0.5285 (3)	0.6834 (5)	0.0628 (15)	
H19	0.5539	0.5364	0.7089	0.075*	
C20	0.4327 (6)	0.6281 (3)	0.4476 (4)	0.0617 (14)	
C21	0.4956 (5)	0.6762 (3)	0.3855 (4)	0.0553 (13)	
C22	0.6181 (6)	0.6895 (4)	0.4194 (5)	0.0657 (15)	
H22	0.6644	0.6678	0.4837	0.079*	
C23	0.6716 (6)	0.7344 (4)	0.3589 (5)	0.0722 (17)	
H23	0.7538	0.7428	0.3828	0.087*	
C24	0.6044 (5)	0.7675 (3)	0.2624 (4)	0.0607 (14)	
C25	0.4824 (6)	0.7539 (3)	0.2282 (4)	0.0669 (15)	
H25	0.4359	0.7749	0.1634	0.080*	
C26	0.4297 (6)	0.7091 (3)	0.2900 (4)	0.0636 (14)	
H26	0.3474	0.7010	0.2665	0.076*	

### Atomic displacement parameters (Å^2^)

**Table:**

	*U*^11^	*U*^22^	*U*^33^	*U*^12^	*U*^13^	*U*^23^
Sn1	0.0800 (4)	0.0953 (4)	0.0957 (4)	0.0001 (3)	0.0303 (3)	0.0250 (3)
O1	0.082 (3)	0.131 (5)	0.105 (4)	−0.013 (3)	0.038 (3)	0.027 (4)
O2	0.089 (3)	0.117 (4)	0.073 (3)	−0.031 (3)	0.008 (3)	0.024 (3)
O3	0.083 (3)	0.100 (3)	0.067 (3)	−0.003 (3)	0.034 (2)	0.021 (2)
N1	0.080 (3)	0.054 (3)	0.054 (3)	−0.003 (2)	0.031 (2)	−0.001 (2)
N2	0.082 (3)	0.057 (3)	0.047 (2)	−0.004 (2)	0.028 (2)	−0.001 (2)
C1	0.141 (10)	0.149 (9)	0.148 (8)	−0.011 (7)	0.046 (8)	0.008 (7)
C2	0.173 (9)	0.149 (10)	0.161 (11)	0.008 (7)	0.048 (8)	−0.004 (8)
C3	0.172 (11)	0.193 (11)	0.200 (10)	0.023 (8)	0.055 (9)	−0.012 (8)
C4	0.200 (13)	0.208 (12)	0.223 (11)	0.026 (9)	0.052 (9)	−0.035 (9)
C5	0.091 (9)	0.109 (8)	0.118 (6)	−0.023 (7)	0.019 (6)	0.010 (6)
C6	0.129 (11)	0.142 (8)	0.134 (6)	0.006 (8)	0.043 (7)	0.007 (6)
C7	0.168 (12)	0.181 (10)	0.154 (7)	0.003 (9)	0.032 (8)	−0.016 (7)
C8	0.191 (12)	0.194 (12)	0.168 (8)	−0.011 (9)	0.030 (8)	−0.001 (8)
C1'	0.141 (10)	0.149 (9)	0.148 (8)	−0.011 (7)	0.046 (8)	0.008 (7)
C2'	0.173 (9)	0.149 (10)	0.161 (11)	0.008 (7)	0.048 (8)	−0.004 (8)
C3'	0.172 (11)	0.193 (11)	0.200 (10)	0.023 (8)	0.055 (9)	−0.012 (8)
C4'	0.200 (13)	0.208 (12)	0.223 (11)	0.026 (9)	0.052 (9)	−0.035 (9)
C5'	0.091 (9)	0.109 (8)	0.118 (6)	−0.023 (7)	0.019 (6)	0.010 (6)
C6'	0.129 (11)	0.142 (8)	0.134 (6)	0.006 (8)	0.043 (7)	0.007 (6)
C7'	0.168 (12)	0.181 (10)	0.154 (7)	0.003 (9)	0.032 (8)	−0.016 (7)
C8'	0.191 (12)	0.194 (12)	0.168 (8)	−0.011 (9)	0.030 (8)	−0.001 (8)
C9	0.089 (5)	0.073 (4)	0.076 (4)	0.001 (4)	0.041 (4)	0.007 (3)
C10	0.102 (6)	0.100 (5)	0.100 (6)	−0.004 (4)	0.058 (5)	0.017 (4)
C11	0.128 (7)	0.081 (5)	0.097 (6)	−0.002 (5)	0.068 (5)	0.016 (4)
C12	0.120 (6)	0.056 (4)	0.066 (4)	0.008 (4)	0.047 (4)	0.005 (3)
C13	0.140 (8)	0.074 (4)	0.077 (5)	0.009 (5)	0.051 (5)	0.014 (4)
C14	0.139 (8)	0.097 (6)	0.074 (5)	0.014 (6)	0.029 (5)	0.030 (4)
C15	0.114 (7)	0.122 (7)	0.081 (5)	0.003 (5)	0.019 (5)	0.024 (5)
C16	0.110 (6)	0.081 (5)	0.072 (4)	−0.004 (4)	0.031 (4)	0.015 (3)
C17	0.105 (6)	0.045 (3)	0.071 (4)	−0.001 (3)	0.044 (4)	−0.004 (3)
C18	0.094 (5)	0.049 (3)	0.058 (3)	−0.001 (3)	0.040 (3)	−0.004 (3)
C19	0.087 (4)	0.051 (3)	0.058 (3)	0.001 (3)	0.033 (3)	−0.001 (3)
C20	0.077 (4)	0.053 (3)	0.054 (3)	−0.002 (3)	0.019 (3)	−0.005 (3)
C21	0.078 (4)	0.046 (3)	0.044 (3)	−0.003 (3)	0.020 (3)	−0.005 (2)
C22	0.070 (4)	0.077 (4)	0.052 (3)	0.008 (3)	0.020 (3)	0.010 (3)
C23	0.070 (4)	0.090 (4)	0.060 (3)	0.004 (3)	0.023 (3)	0.014 (3)
C24	0.074 (4)	0.063 (3)	0.051 (3)	0.002 (3)	0.026 (3)	0.000 (3)
C25	0.088 (5)	0.063 (3)	0.047 (3)	−0.002 (3)	0.015 (3)	0.006 (3)
C26	0.071 (4)	0.065 (3)	0.053 (3)	−0.008 (3)	0.014 (3)	−0.001 (3)

### Geometric parameters (Å, °)

**Table:**

Sn1—C5	2.02 (2)	C3'—H3'B	0.9700
Sn1—C1	2.04 (2)	C4'—H4'A	0.9600
Sn1—O1	2.076 (5)	C4'—H4'B	0.9600
Sn1—O2	2.131 (5)	C4'—H4'C	0.9600
Sn1—N1	2.148 (5)	C5'—C6'	1.536 (10)
Sn1—C1'	2.16 (2)	C5'—H5'A	0.9700
Sn1—C5'	2.28 (2)	C5'—H5'B	0.9700
O1—C9	1.301 (8)	C6'—C7'	1.522 (10)
O2—C20	1.270 (7)	C6'—H6'A	0.9700
O3—C24	1.348 (7)	C6'—H6'B	0.9700
O3—H3	0.8400	C7'—C8'	1.537 (10)
N1—C19	1.300 (7)	C7'—H7'A	0.9700
N1—N2	1.404 (6)	C7'—H7'B	0.9700
N2—C20	1.312 (7)	C8'—H8'A	0.9600
C1—C2	1.518 (10)	C8'—H8'B	0.9600
C1—H1A	0.9700	C8'—H8'C	0.9600
C1—H1B	0.9700	C9—C18	1.397 (10)
C2—C3	1.554 (10)	C9—C10	1.433 (10)
C2—H2A	0.9700	C10—C11	1.344 (11)
C2—H2B	0.9700	C10—H10	0.9300
C3—C4	1.549 (10)	C11—C12	1.422 (11)
C3—H3A	0.9700	C11—H11	0.9300
C3—H3B	0.9700	C12—C13	1.396 (10)
C4—H4A	0.9600	C12—C17	1.407 (9)
C4—H4B	0.9600	C13—C14	1.325 (12)
C4—H4C	0.9600	C13—H13	0.9300
C5—C6	1.526 (10)	C14—C15	1.384 (12)
C5—H5A	0.9700	C14—H14	0.9300
C5—H5B	0.9700	C15—C16	1.362 (11)
C6—C7	1.532 (10)	C15—H15	0.9300
C6—H6A	0.9700	C16—C17	1.384 (11)
C6—H6B	0.9700	C16—H16	0.9300
C7—C8	1.546 (10)	C17—C18	1.457 (9)
C7—H7A	0.9700	C18—C19	1.422 (8)
C7—H7B	0.9700	C19—H19	0.9300
C8—H8A	0.9600	C20—C21	1.488 (8)
C8—H8B	0.9600	C21—C26	1.375 (8)
C8—H8C	0.9600	C21—C22	1.387 (8)
C1'—C2'	1.517 (10)	C22—C23	1.372 (8)
C1'—H1'A	0.9700	C22—H22	0.9300
C1'—H1'B	0.9700	C23—C24	1.390 (8)
C2'—C3'	1.562 (10)	C23—H23	0.9300
C2'—H2'A	0.9700	C24—C25	1.383 (9)
C2'—H2'B	0.9700	C25—C26	1.377 (8)
C3'—C4'	1.523 (10)	C25—H25	0.9300
C3'—H3'A	0.9700	C26—H26	0.9300
			
C5—Sn1—C1	101.7 (10)	C4'—C3'—H3'A	110.0
C5—Sn1—O1	94.2 (6)	C2'—C3'—H3'A	110.0
C1—Sn1—O1	97.8 (7)	C4'—C3'—H3'B	110.0
C5—Sn1—O2	98.7 (6)	C2'—C3'—H3'B	110.0
C1—Sn1—O2	100.2 (8)	H3'A—C3'—H3'B	108.4
O1—Sn1—O2	155.2 (2)	C3'—C4'—H4'A	109.5
C5—Sn1—N1	128.7 (7)	C3'—C4'—H4'B	109.5
C1—Sn1—N1	129.6 (6)	H4'A—C4'—H4'B	109.5
O1—Sn1—N1	82.56 (19)	C3'—C4'—H4'C	109.5
O2—Sn1—N1	72.85 (18)	H4'A—C4'—H4'C	109.5
C5—Sn1—C1'	123.4 (10)	H4'B—C4'—H4'C	109.5
C1—Sn1—C1'	23.5 (8)	C6'—C5'—Sn1	101.6 (13)
O1—Sn1—C1'	103.1 (6)	C6'—C5'—H5'A	111.5
O2—Sn1—C1'	87.3 (6)	Sn1—C5'—H5'A	111.5
N1—Sn1—C1'	107.0 (7)	C6'—C5'—H5'B	111.5
C5—Sn1—C5'	13.5 (11)	Sn1—C5'—H5'B	111.5
C1—Sn1—C5'	112.9 (8)	H5'A—C5'—H5'B	109.3
O1—Sn1—C5'	99.5 (6)	C7'—C6'—C5'	111.1 (9)
O2—Sn1—C5'	89.1 (7)	C7'—C6'—H6'A	109.4
N1—Sn1—C5'	116.7 (6)	C5'—C6'—H6'A	109.4
C1'—Sn1—C5'	132.7 (9)	C7'—C6'—H6'B	109.4
C9—O1—Sn1	134.0 (4)	C5'—C6'—H6'B	109.4
C20—O2—Sn1	115.1 (4)	H6'A—C6'—H6'B	108.0
C24—O3—H3	109.5	C6'—C7'—C8'	110.4 (10)
C19—N1—N2	115.3 (5)	C6'—C7'—H7'A	109.6
C19—N1—Sn1	128.7 (4)	C8'—C7'—H7'A	109.6
N2—N1—Sn1	116.0 (3)	C6'—C7'—H7'B	109.6
C20—N2—N1	111.0 (5)	C8'—C7'—H7'B	109.6
C2—C1—Sn1	112.8 (13)	H7'A—C7'—H7'B	108.1
C2—C1—H1A	109.0	C7'—C8'—H8'A	109.5
Sn1—C1—H1A	109.0	C7'—C8'—H8'B	109.5
C2—C1—H1B	109.0	H8'A—C8'—H8'B	109.5
Sn1—C1—H1B	109.0	C7'—C8'—H8'C	109.5
H1A—C1—H1B	107.8	H8'A—C8'—H8'C	109.5
C1—C2—C3	109.5 (10)	H8'B—C8'—H8'C	109.5
C1—C2—H2A	109.8	O1—C9—C18	125.0 (6)
C3—C2—H2A	109.8	O1—C9—C10	115.7 (7)
C1—C2—H2B	109.8	C18—C9—C10	119.3 (7)
C3—C2—H2B	109.8	C11—C10—C9	120.5 (8)
H2A—C2—H2B	108.2	C11—C10—H10	119.7
C4—C3—C2	108.0 (9)	C9—C10—H10	119.7
C4—C3—H3A	110.1	C10—C11—C12	122.5 (7)
C2—C3—H3A	110.1	C10—C11—H11	118.7
C4—C3—H3B	110.1	C12—C11—H11	118.7
C2—C3—H3B	110.1	C13—C12—C11	120.8 (7)
H3A—C3—H3B	108.4	C13—C12—C17	120.6 (8)
C3—C4—H4A	109.5	C11—C12—C17	118.6 (7)
C3—C4—H4B	109.5	C14—C13—C12	120.9 (7)
H4A—C4—H4B	109.5	C14—C13—H13	119.5
C3—C4—H4C	109.5	C12—C13—H13	119.5
H4A—C4—H4C	109.5	C13—C14—C15	119.2 (8)
H4B—C4—H4C	109.5	C13—C14—H14	120.4
C6—C5—Sn1	116.3 (13)	C15—C14—H14	120.4
C6—C5—H5A	108.2	C16—C15—C14	121.7 (10)
Sn1—C5—H5A	108.2	C16—C15—H15	119.2
C6—C5—H5B	108.2	C14—C15—H15	119.2
Sn1—C5—H5B	108.2	C15—C16—C17	120.6 (7)
H5A—C5—H5B	107.4	C15—C16—H16	119.7
C5—C6—C7	111.4 (10)	C17—C16—H16	119.7
C5—C6—H6A	109.3	C16—C17—C12	117.0 (7)
C7—C6—H6A	109.3	C16—C17—C18	123.7 (6)
C5—C6—H6B	109.3	C12—C17—C18	119.3 (7)
C7—C6—H6B	109.3	C9—C18—C19	121.4 (6)
H6A—C6—H6B	108.0	C9—C18—C17	119.7 (6)
C6—C7—C8	108.3 (9)	C19—C18—C17	118.9 (6)
C6—C7—H7A	110.0	N1—C19—C18	128.1 (6)
C8—C7—H7A	110.0	N1—C19—H19	116.0
C6—C7—H7B	110.0	C18—C19—H19	116.0
C8—C7—H7B	110.0	O2—C20—N2	124.1 (6)
H7A—C7—H7B	108.4	O2—C20—C21	116.8 (5)
C7—C8—H8A	109.5	N2—C20—C21	119.1 (5)
C7—C8—H8B	109.5	C26—C21—C22	118.2 (5)
H8A—C8—H8B	109.5	C26—C21—C20	118.7 (5)
C7—C8—H8C	109.5	C22—C21—C20	123.0 (5)
H8A—C8—H8C	109.5	C23—C22—C21	120.7 (5)
H8B—C8—H8C	109.5	C23—C22—H22	119.7
C2'—C1'—Sn1	115.2 (14)	C21—C22—H22	119.7
C2'—C1'—H1'A	108.5	C22—C23—C24	120.8 (6)
Sn1—C1'—H1'A	108.5	C22—C23—H23	119.6
C2'—C1'—H1'B	108.5	C24—C23—H23	119.6
Sn1—C1'—H1'B	108.5	O3—C24—C25	122.5 (5)
H1'A—C1'—H1'B	107.5	O3—C24—C23	118.9 (6)
C1'—C2'—C3'	108.8 (10)	C25—C24—C23	118.6 (5)
C1'—C2'—H2'A	109.9	C26—C25—C24	120.0 (5)
C3'—C2'—H2'A	109.9	C26—C25—H25	120.0
C1'—C2'—H2'B	109.9	C24—C25—H25	120.0
C3'—C2'—H2'B	109.9	C21—C26—C25	121.7 (6)
H2'A—C2'—H2'B	108.3	C21—C26—H26	119.1
C4'—C3'—C2'	108.5 (10)	C25—C26—H26	119.1
			
C5—Sn1—O1—C9	123.5 (10)	O2—Sn1—C5'—C6'	−71.1 (14)
C1—Sn1—O1—C9	−134.0 (9)	N1—Sn1—C5'—C6'	−141.4 (13)
O2—Sn1—O1—C9	2.1 (11)	C1'—Sn1—C5'—C6'	14 (2)
N1—Sn1—O1—C9	−4.9 (7)	Sn1—C5'—C6'—C7'	−172.0 (14)
C1'—Sn1—O1—C9	−110.7 (10)	C5'—C6'—C7'—C8'	−64 (3)
C5'—Sn1—O1—C9	111.0 (9)	Sn1—O1—C9—C18	3.2 (12)
C5—Sn1—O2—C20	−136.3 (9)	Sn1—O1—C9—C10	−175.8 (5)
C1—Sn1—O2—C20	120.0 (8)	O1—C9—C10—C11	−180.0 (7)
O1—Sn1—O2—C20	−15.7 (9)	C18—C9—C10—C11	1.0 (11)
N1—Sn1—O2—C20	−8.4 (5)	C9—C10—C11—C12	−1.7 (12)
C1'—Sn1—O2—C20	100.3 (8)	C10—C11—C12—C13	−177.0 (7)
C5'—Sn1—O2—C20	−126.8 (7)	C10—C11—C12—C17	0.2 (11)
C5—Sn1—N1—C19	−86.0 (9)	C11—C12—C13—C14	177.6 (8)
C1—Sn1—N1—C19	97.4 (11)	C17—C12—C13—C14	0.3 (11)
O1—Sn1—N1—C19	3.5 (5)	C12—C13—C14—C15	−0.6 (13)
O2—Sn1—N1—C19	−173.4 (5)	C13—C14—C15—C16	0.4 (14)
C1'—Sn1—N1—C19	105.0 (8)	C14—C15—C16—C17	0.1 (13)
C5'—Sn1—N1—C19	−93.4 (8)	C15—C16—C17—C12	−0.4 (10)
C5—Sn1—N1—N2	95.5 (8)	C15—C16—C17—C18	−179.4 (7)
C1—Sn1—N1—N2	−81.1 (10)	C13—C12—C17—C16	0.1 (9)
O1—Sn1—N1—N2	−175.0 (4)	C11—C12—C17—C16	−177.2 (6)
O2—Sn1—N1—N2	8.1 (3)	C13—C12—C17—C18	179.2 (6)
C1'—Sn1—N1—N2	−73.5 (7)	C11—C12—C17—C18	1.9 (9)
C5'—Sn1—N1—N2	88.1 (8)	O1—C9—C18—C19	2.0 (10)
C19—N1—N2—C20	174.5 (5)	C10—C9—C18—C19	−179.1 (6)
Sn1—N1—N2—C20	−6.8 (5)	O1—C9—C18—C17	−177.8 (6)
C5—Sn1—C1—C2	168 (2)	C10—C9—C18—C17	1.2 (9)
O1—Sn1—C1—C2	72 (2)	C16—C17—C18—C9	176.4 (6)
O2—Sn1—C1—C2	−91 (2)	C12—C17—C18—C9	−2.6 (8)
N1—Sn1—C1—C2	−14 (2)	C16—C17—C18—C19	−3.4 (9)
C1'—Sn1—C1—C2	−33 (2)	C12—C17—C18—C19	177.7 (5)
C5'—Sn1—C1—C2	176.1 (19)	N2—N1—C19—C18	177.9 (5)
Sn1—C1—C2—C3	−175 (2)	Sn1—N1—C19—C18	−0.6 (8)
C1—C2—C3—C4	156 (3)	C9—C18—C19—N1	−3.2 (9)
C1—Sn1—C5—C6	86.3 (19)	C17—C18—C19—N1	176.6 (5)
O1—Sn1—C5—C6	−174.9 (17)	Sn1—O2—C20—N2	8.3 (8)
O2—Sn1—C5—C6	−16.1 (19)	Sn1—O2—C20—C21	−172.3 (4)
N1—Sn1—C5—C6	−91.0 (18)	N1—N2—C20—O2	−1.0 (8)
C1'—Sn1—C5—C6	76 (2)	N1—N2—C20—C21	179.6 (4)
C5'—Sn1—C5—C6	−61 (4)	O2—C20—C21—C26	8.6 (8)
Sn1—C5—C6—C7	−179 (2)	N2—C20—C21—C26	−172.0 (5)
C5—C6—C7—C8	171 (3)	O2—C20—C21—C22	−171.4 (6)
C5—Sn1—C1'—C2'	110 (2)	N2—C20—C21—C22	8.0 (8)
C1—Sn1—C1'—C2'	85 (3)	C26—C21—C22—C23	0.0 (9)
O1—Sn1—C1'—C2'	6(2)	C20—C21—C22—C23	−179.9 (5)
O2—Sn1—C1'—C2'	−151 (2)	C21—C22—C23—C24	−0.1 (9)
N1—Sn1—C1'—C2'	−80 (2)	C22—C23—C24—O3	−179.5 (5)
C5'—Sn1—C1'—C2'	123 (2)	C22—C23—C24—C25	−0.3 (9)
Sn1—C1'—C2'—C3'	172.6 (15)	O3—C24—C25—C26	179.9 (5)
C1'—C2'—C3'—C4'	111 (3)	C23—C24—C25—C26	0.8 (9)
C5—Sn1—C5'—C6'	64 (4)	C22—C21—C26—C25	0.5 (8)
C1—Sn1—C5'—C6'	29.6 (18)	C20—C21—C26—C25	−179.6 (5)
O1—Sn1—C5'—C6'	132.2 (14)	C24—C25—C26—C21	−0.9 (9)

### Hydrogen-bond geometry (Å, °)

**Table:**

*D*—H···*A*	*D*—H	H···*A*	*D*···*A*	*D*—H···*A*
O3—H3···N2^i^	0.84	1.99	2.824 (6)	177

Symmetry codes: (i) *x*, −*y*+3/2, *z*−1/2.
